# Impact of Pelvic Fracture on Patients with Blunt Bowel Mesenteric Injury: Is Immediate Laparotomy Warranted?

**DOI:** 10.3390/life14010016

**Published:** 2023-12-21

**Authors:** Ting-Min Hsieh, Po-Chun Chuang, Chun-Ting Liu, Bei-Yu Wu, Chien-Hung Wu, Fu-Jen Cheng

**Affiliations:** 1 Division of Trauma, Department of Surgery, Kaohsiung Chang Gung Memorial Hospital, Chang Gung University College of Medicine, Kaohsiung 833, Taiwan; hs168hs168@gmail.com; 2 Department of Emergency Medicine, Kaohsiung Chang Gung Memorial Hospital, Chang Gung University College of Medicine, Kaohsiung 833, Taiwan; bogy1102@cgmh.org.tw; 3 Department of Chinese Medicine, Kaohsiung Chang Gung Memorial Hospital, Chang Gung University College of Medicine, Kaohsiung 833, Taiwan; juntin0214@cgmh.org.tw (C.-T.L.); y7802@cgmh.org.tw (B.-Y.W.); 4 Department of Emergency Medicine, Yunlin Chang Gung Memorial Hospital, Chang Gung University College of Medicine, Yunlin 638, Taiwan

**Keywords:** blunt abdominal trauma, blunt bowel mesenteric injuries, pelvic fracture, emergency department, laparotomy

## Abstract

The management of blunt abdominopelvic trauma with combined hemoperitoneum and pelvic fractures is challenging for trauma surgeons. Although angioembolization can achieve hemostasis in most visceral organ injuries and pelvic fractures after blunt abdominal trauma, it cannot effectively control hemorrhage in patients with blunt bowel mesenteric injury (BBMI). This study aimed to determine the risk factors associated with hemodynamically unstable patients with BBMI and to test the hypothesis that pelvic fracture is an independent risk factor for patients with unstable BBMI and concomitant pelvic fracture to guide the therapeutic sequence for difficult-to-manage patients. This retrospective study reviewed the data of hospitalized patients with trauma between 2009 and 2021 and included 158 adult patients with surgically proven BBMI. The patients were divided on the basis of the presence of a shock episode before emergency laparotomy. The shock group included 44.3% of all patients in the study (n = 70). Clinical injury severity and prognosis for patients in the shock group were poorer than those for patients in the non-shock group, and more invasive treatments and transfusions were performed for patients in the shock group than for those in the non-shock group. Pelvic fractures were more frequently associated with the shock group than with the non-shock group (21.4% vs. 5.7%; *p* = 0.003). In multivariate analysis, the presence of intracerebral hemorrhage (odds ratio [OR] = 10.87, 95% confidence intervals [CIs]: 1.70–69.75) and rib fracture (OR = 5.94, 95% CIs = 1.06–33.45) was identified as an independent predictor of shock, whereas the effect of pelvic fracture did not achieve statistical significance (OR = 2.94, 95% CIs = 0.66–13.13) after adjusting for confounding factors. For patients with BBMI, outcomes need to be improved during early diagnosis, and treatments should be expeditiously performed on the basis of the rapid identification of unstable hemodynamic status. Our results support the recommendation of emergency laparotomy in unstable patients with concomitant pelvic fractures, followed by damage control TAE if needed.

## 1. Introduction

Despite advances in trauma surgery and care, blunt abdominopelvic injuries remain challenging for trauma surgeons, particularly in the face of hemodynamic instability (HI). In addition to intraperitoneal bleeding from blunt abdominal trauma (BAT), retroperitoneal bleeding should be considered in unstable patients with concomitant pelvic fracture (PF). Similarly, patients with any PF may experience a concomitant intra-abdominal injury from 16% to 42%, which has increased from 62% to 77% in patients with concomitant retroperitoneal hemorrhage and 70% in unstable patients, respectively [1,2,3,4,5]. PF also results in approximately 7% to 13% retroperitoneal hemorrhages requiring emergency interventions and 7.6% to 55% HI [3,4,6,7,8,9], contributing to a mortality rate of 4.3% to 19%, and even up to 31% to 46% in unstable patients [4,5,6,8,10,11]. Accordingly, it is difficult to rapidly determine the bleeding source and make decisions in a short period under stressful situations involving HI. It is a good thing that in the era of non-operative management (NOM), transcatheter arterial embolization (TAE) can achieve hemostasis in most solid organ injuries following BAT and PF [3,8,9,12]. However, it is noteworthy that intra-abdominal injury is easily overlooked in unstable BAT patients with concomitant PF who had received TAE first for hemostasis before computed tomography (CT) examination, in whom blunt bowel mesenteric injuries (BBMIs) accounted for 86% of missed injuries [7]. Given the enthusiasm for NOM, potential shock, regardless of mesentery tear or sepsis as a result of bowel injuries, has been proved to be not only the most common indication for laparotomy but also a reason for NOM failure in BAT patients [7,12]. Although the widespread use of CT enhances the success rate of NOM, the high false-negative rate of CT and the nonspecific clinical presentation in BBMI often lead to delayed management, which increases morbidity and mortality [13,14,15,16,17,18]. However, the CT findings of combined hemopneumoperitoneum and PF in patients with unstable BAT clinically aggravate the management dilemma. 

Although numerous publications have established protocol-based algorithm implantation with cavity hemorrhage management in life-threatening patients with PF [8], the therapeutic strategy and sequence of pre-peritoneal packing or TAE for PF still vary in different trauma centers because of the resources available in clinical practice [1,11,19,20]. Clinical studies [19] have reported that hemodynamically unstable BAT patients with concomitant intraperitoneal and retroperitoneal hemorrhage can be treated with post-laparotomy TAE; PF is a useful predictor for this clinical practice. Thorson et al. supported this proposal and suggested that laparotomy should take priority over TAE in patients with hemodynamically unstable PF [11]. By contrast, Eastridge et al. considered that in patients in shock with unstable type PF, TAE should be arranged before laparotomy, even in the presence of hemoperitoneum [1]. Other studies [20] supported these treatment strategies and reported that difficult-to-manage patients with PF and hemoperitoneum had the same mortality rate regardless of hemodynamic status or initial treatment sequence (laparotomy first or TAE first). Nevertheless, the abovementioned populations estimated in these studies usually included overall organ injuries within the abdomen (either solid organs or hollow organs) after BAT; the discussion on the context between BBMI necessitating laparotomy and PF is limited.

Although BBMI is the third vulnerable organ after BAT, followed by the liver and spleen, its incidence only accounts for approximately 0.3% to 0.6% of total trauma admissions and 4% to 4.7% of BAT admissions in Taiwan trauma centers [16,21]. Given the high dependence on CT use, rarity of BBMI, and preference for NOM, the challenge for trauma surgeons in the face of surgical BBMI patients continues to evolve. These challenges are further exacerbated by unstable patients with BBMI and concurrent PF. To the best of our knowledge, no study has documented the association between surgical BBMI and HI after considering associated injuries. There is a need to assist with decision making for an optimal strategy early, such that appropriate treatment may be expeditiously administered to unstable patients with BBMI and PF. The objectives of this study were as follows: (1) to investigate the clinical presentation, associated injuries, and outcomes that might correlate with unstable patients with surgical BBMI and (2) to analyze the effect of concurrent PF in the determinants of patients sustaining HI following surgical BBMI to guide the therapeutic sequence.

## 2. Materials and Methods

### 2.1. Ethics Statement

This study was approved by the Institutional Review Board (IRB) of Chang Gung Memorial Hospital (approval number: 201902275B0). The need for informed consent was waived according to IRB regulations.

### 2.2. Study Population

This retrospective study reviewed all data added to the Trauma Registry System from 1 January 2009 to 31 December 2021 in a 2686-bed facility and Level I regional trauma center that provides care to trauma patients in southern Taiwan. All data were prospectively collected from the medical records of hospitalized patients with trauma and retrospectively analyzed. The patients enrolled in this study were adult trauma patients (>16 years of age) who underwent emergency therapeutic laparotomy for suspected BBMI. During the 13-year investigation period, only patients with BBMI and surgically proven gastrointestinal tract or mesenteric injury were recruited. Patients with isolated stomach, duodenal, or rectal injuries were included. Therapeutic laparotomy was defined as the use of procedures to repair or resect the bowel or control active bleeding. Finally, the included patients were categorized into two groups on the basis of the presence of shock episodes during the period between arrival at the initial emergency department (ED) and emergency laparotomy. Patients with a systolic blood pressure less than 90 mmHg or even greater than 90 remained to need persistent fluid resuscitation or transfusion to maintain were defined as hemodynamic instability. Patients with a history of shock before laparotomy were compared with patients without a history of shock episodes, and associated injuries were assessed for their ability to predict the presence of shock.

### 2.3. Study Parameters

The following variables were extracted for each patient: demographic data such as age and sex; clinical and trauma data such as injury severity score (ISS), new ISS (NISS), trauma resuscitation ISS (TRISS), revised trauma score (RTS), and abbreviated injury score (AIS) over the head, face, chest, abdomen, and extremities; vital signs at the ED, including SBP, heart rate, respiratory rate, and Glasgow Coma Scale score; injury mechanisms; clinical presentation such as hemoglobin level upon arrival at the ED, incidence of intubation, and tube thoracostomy at the ED; status of blood transfusion (BT), including the incidence of BT at the ED and massive transfusion; amount of packed red blood cells and fresh frozen plasma transfused at the ED within 24 h and at the operating room and ward; and operative findings, including the incidence of isolated small bowel injury (defined as gastric, duodenum, or small bowel injury, including ischemia, rupture, serosa injury, or hematoma), isolated colon injury (defined as colon or rectum injury, including ischemia, rupture, serosa injury, or hematoma), isolated mesentery injury (defined as mesentery injury, including ischemia, rupture, serosa injury, or hematoma), combined injury (defined as either small bowel or colon injury concomitant with mesenteric injury, including ischemia, rupture, serosa injury, or hematoma), and operative blood loss and delayed operation (defined as patients whose emergency laparotomy was performed during admission to the intensive care unit (ICU) or ward under a miss diagnosis rather than initially at the ED). Outcome data: Morbidity and mortality were classified by cause as either caused by bowel injury or bleeding injury, 24 h mortality, and length of stay in the hospital and ICU. Morbidities were identified during chart reviews on the basis of standard definitions. Bowel-related mortality was defined as mortality due to abdomen-related sepsis following surgery. Exsanguination-related mortality was defined as mortality due to surgically proven hemorrhagic shock resulting from bowel or mesenteric bleeding. Overall morbidities included sepsis, pneumonia, septic shock, unplanned intubation, intra-abdominal abscess, leakage, coagulopathy, acute kidney injury, acidosis, urinary tract infection, stroke, pulmonary embolism, acute respiratory distress syndrome, pleural effusion, enterocutaneous fistula, wound infection, wound dehiscence, abdominal compartment, tracheostomy, extracorporeal membrane oxygenation (ECMO), return to the operating room, and hemodialysis.

### 2.4. Statistical Analysis

The data were analyzed using IBM SPSS Statistics for Windows, version 20.0 (IBM Corp., Armonk, NY, USA). Continuous variables, including age, blood pressure, heart rate, and hospital length of stay, were reported as medians and interquartile ranges. Considering the potential impact of a small sample size on our statistical analysis, we treated the data as non-normally distributed. The Mann–Whitney U test was therefore employed to analyze these continuous variables. In the initial phase, factors such as age, sex, associated injuries, and BBMI injury pattern based on intra-operative findings were identified as significant in univariate analysis. These were subsequently incorporated into a binary regression model to identify independent predictors of shock episodes, allowing for the adjustment of potential confounders. We were particularly mindful of the risk of overfitting in the regression model and the limited power in detecting significant differences due to the smaller sample size. To analyze the temporal relationship between shock and morbidity, Kaplan–Meier analysis was utilized, and the log-rank test was applied to compare the morbidity and mortality curves between the shock and non-shock groups. The threshold for statistical significance was set at *p* < 0.05.

## 3. Results

### 3.1. Patient Characteristics, Clinical Presentation, and Outcome

**Figure 1:**
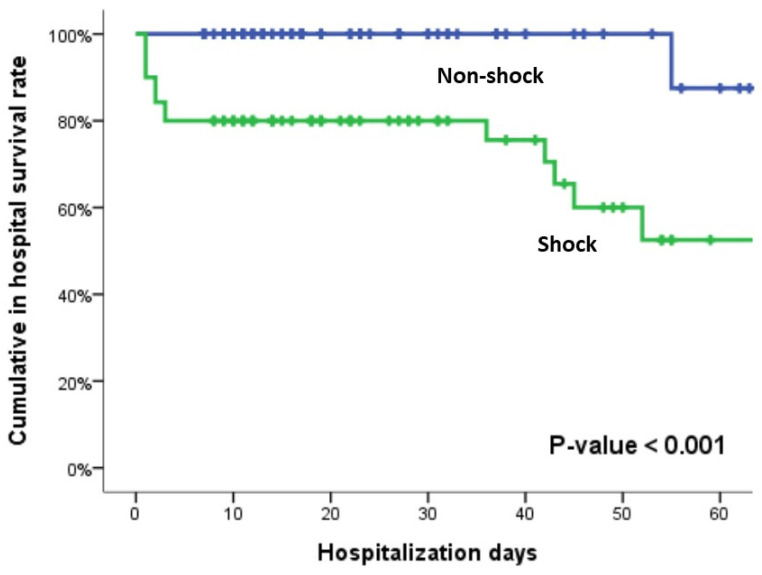
In-hospital survival rate. Kaplan–Meier curves displaying in-hospital survival for patients who experienced surgical BBMI in shock and non-shock groups.

After a detailed chart review, seven patients were excluded, including those aged <16 years (n = 2) and those who underwent non-therapeutic laparotomy (n = 5). One hundred and fifty-eight patents meeting the study criteria for surgically proven BBMI were initially treated in the ED and subsequently admitted to the ward or ICU. Among the 158 patients, gastric injury (n = 1) and duodenal injury (n = 4) were classified as bowel injury types, whereas rectal injury (n = 2) was classified as a colon injury type. When comparing the non-shock group with the shock group, the shock group had more severe anatomic injuries due to ISS (9 vs. 21.5, *p* < 0.001) and NISS (13 vs. 27, *p* < 0.001); physiological injuries due to RTS (7.84 vs. 7.108, *p* < 0.001); probability of survival due to TRISS (0.99 vs. 0.97, *p* < 0.001); worse ED vital signs; a more critical ED clinical presentation; and a greater percentage of transfusion at ED. Regarding the intra-operative findings, the non-shock group had a significantly higher incidence of isolated bowel injury (*p* < 0.001) and isolated colon injury (*p* = 0.012) than the shock group, whereas the shock group had a significantly higher incidence of isolated mesenteric injury (*p* < 0.001) or combined injury (*p* = 0.004) and a significantly greater amount of operative blood loss (*p* < 0.001) than the non-shock group. Note that the non-shock group had a significantly higher frequency of delayed operations compared with the shock group (17% vs. 4.3%, *p* = 0.012). Additionally, overall morbidity and mortality were greater in the shock group than in the non-shock group (90% vs. 48.9% [*p* < 0.001] and 27.1% vs. 2.3% [*p* < 0.001], respectively) (Table 1). On the basis of the results of the Kaplan–Meier analysis, patients in shock had a higher and earlier mortality rate than those who were not in shock (*p* < 0.001) (Figure 1).

### 3.2. Injury Severity and Injury Pattern

The distribution of AIS injuries in each body region in the two groups is shown in Table 2. The shock group had significantly higher injury severity over AIS of the head (*p* < 0.001), chest (*p* = 0.05), abdomen (*p* < 0.001), and extremities (*p* = 0.014) than the non-shock group. Furthermore, the shock group had more frequency in AIS head ≥ 2 (*p* < 0.001), AIS head ≥ 3 (*p* < 0.001), AIS chest ≥ 3 (*p* = 0.034), and AIS extremities ≥ 2 (*p* = 0.006) compared with the shock (-) group.

The most commonly associated injured organs were the liver (18.4%), low limb fractures (17.7%), rib fractures (17.1%), and hemopneumothorax (17.1%). PFs were significantly more frequent in the shock group than in the non-shock group (21.4% vs. 12.7%, *p* = 0.003). A comparison of the incidence of each specific injury between the two groups is presented in Table 3.

### 3.3. Predicting Factors of Shock Episode

Univariate and subsequent multivariate analyses identified the injury-related risk factors associated with shock episodes (Table 4). Univariate analyses revealed that vessel injury (*p* = 0.011), intracerebral hemorrhage (ICH) (*p* = 0.001), rib fracture (*p* < 0.001), PF (*p* = 0.006), and lower limb fracture (*p* = 0.022) were associated with an increased risk of shock. Multivariate analyses revealed that only ICH (OR = 10.87, 95% confidence interval [CI] = 1.70–69.75) and rib fracture (OR = 5.94, 95% CI = 1.06–33.45) were independently associated with the presence of shock. The effect of PF (OR = 2.94, 95% CI 0.66–13.13) during a shock episode was not statistically significant after adjusting the confounders. Furthermore, it is expected that isolated mesentery injury (OR = 23.5, 95% CI = 4.61–119.88) and combined injury (OR = 25.43, 95% CI = 4.89–132.28) are strongly independent predictors of shock.

### 3.4. Overall Morbidities

**Figure 2:**
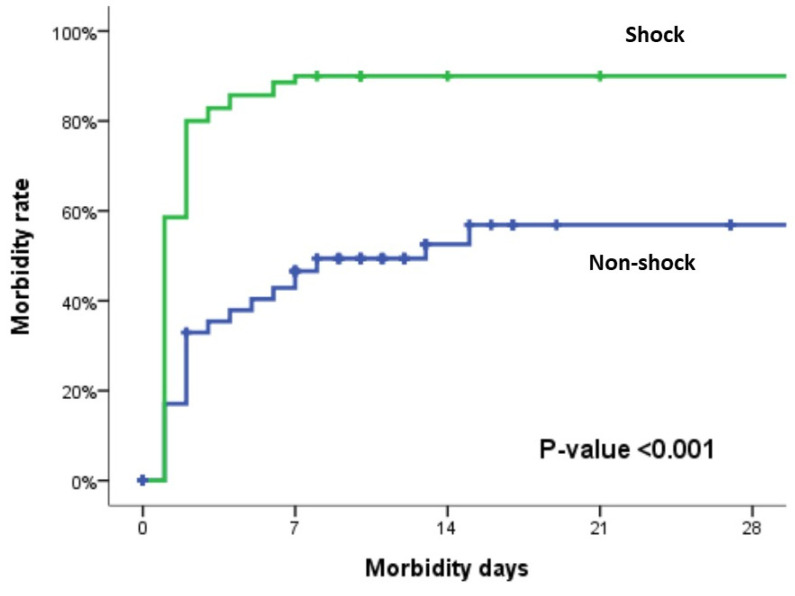
Kaplan–Meier curves displaying in-hospital morbidity for patients who experienced surgical BBMI in shock-positive and shock-negative groups.

Morbidity rates are shown in Table 5. There were no significant differences in the overall morbidity rates between the groups, except for unplanned ventilation (*p* < 0.001), coagulopathy (*p* < 0.001), acute renal failure (*p* < 0.001), acidosis (*p* < 0.001), stroke (*p* = 0.023), abdominal compartment syndrome (*p* = 0.045), and ECMO intervention (*p* = 0.037), which were significantly more common in the shock group than in the non-shock group. Additionally, according to the results of the Kaplan–Meier analysis, patients in the shock-positive group had a significantly higher and earlier rate of complications compared with those in shock-negative patients (*p* < 0.001) (Figure 2).

## 4. Discussion

In our series, we analyzed a cohort of patients with surgically proven BBMI and compared the clinical presence of shock episodes in patients with BBMI to determine the risk factors associated with sustaining an unstable hemodynamic status and to assess whether PF was an independent factor in guiding the therapeutic sequence. Our study suggests PF is significantly more likely in unstable BBMI patients, but significance diminishes when controlling for confounders. This result might suggest that PF was not the primary cause of HI in unstable BBMI patients. Therefore, emergency laparotomy is recommended as the initial approach, with the subsequent consideration of TAE to control bleeding from the pelvic fracture. 

In the current study, particularly remarkable was the fact that near half (44%) of the patients who had a hypotension episode in the ED or ICU before surgery had a median ISS of 21.5 and accounted for 65% (55/85) of ISS ≥ 16 and 78% (32/41) of ISS ≥ 25, respectively, thus underscoring the severity of injury in unstable patients with surgical BBMI. Our results showed that patients with shock had worse clinical presentation and increased morbidity and mortality. This finding is also supported by studies [22] that reported that a stable hemodynamic status was related to significantly fewer complications and better outcomes; however, this is not in agreement with the study of Al-Hassani [15]. In addition, some authors believe that given the advances in resuscitation and ICU care, the effect of hemodynamics on the outcomes in patients with BBMI has decreased, and it is not a significant predictor for surgical intervention. Instead, they considered the time to surgery to be a key component for better prognosis and adaptable determinants [23,24]. Malinoski et al. [14] retrospectively studied 195 patients with blunt bowel injuries and concluded that patients who had a delay of more than 5 h between ED arrival and laparotomy had 3.2 times the odds of mortality. Meanwhile, in another multicenter study, Fakhry et al. [13] retrospectively studied 198 patients with blunt intestinal injuries and recommended that even a short delay of less than 8 h in the diagnosis of this injury would increase morbidity and mortality. They reported that, regarding isolated bowel injuries, patients who had a delayed diagnosis had 19.3 times the odds of mortality than those who had a prompt diagnosis. Interestingly, a unique finding of our study is that although previous studies have reported poor prognosis in delayed operation, our results showed a significant decrease in the incidence of delayed operation in patients with shock, thus implying that the patient’s unstable hemodynamic status could attract us to acquire more attention and early intervention. Our observation is consistent with that reported by Okishio et al. [24], in which BBMI patients with HI had a significantly shorter time interval between ED admission and surgical intervention than stable patients (109 min vs. 191 min, *p* = 0.0009). Similarly, this finding is supported by the study by Al-Hassani et al. [15], in which patients with hypotension were significantly more frequent in the group surgically treated within 8 h than in the group treated after 8 h. Conversely, this finding is inconsistent with the study of Hong et al. [25] in evaluating the outcomes of the delay in surgical intervention for blunt small bowel injuries. They found that patients did not have a significantly different percentage of shock in comparison with either three groups (≤8, 8–24, and >24 h) or two groups (≤24 and >24 h). Our results also indicated that BBMI is more easily underestimated in patients with stable hemodynamics after BAT. Although our study did not directly explore the causality between delayed operation and HI, further investigations into the association between shock and the time from admission to surgery are needed. 

Concerning associated injuries, BBMI is common in polytrauma patients, accounting for over half in a BBMI cohort, influencing clinical presentations [13,15]. Our findings reveal a higher frequency of hemodynamically unstable patients compared to stable ones, suggesting that PF is often linked to life-threatening blunt abdominal trauma (BAT). The complexity of abdominopelvic trauma is exacerbated by the challenge of the timely identification of concurrent intra-abdominal injuries, with BBMI representing only 4.4% of those with PF [5]. A prior study using the Japan Trauma Data Bank (JTDB) reported that among blunt trauma patients with PF and hemoperitoneum undergoing laparotomy or transcatheter arterial embolization (TAE) as initial interventions, more than half exhibited hollow organ injuries (HI), while only 18% were associated with BBMI-related injuries [20]. Fu et al. [7], in a retrospective study of unstable PF patients initially treated with TAE for hemostasis without CT scans, found that 36% of patients required post-TAE laparotomy due to clinical deterioration, with 86% associated with surgical BBMI. They emphasized the risk of overlooking such injuries and highlighted the importance of early CT imaging post-hemodynamic stabilization. A previous study [15], assessing predictors of surgical BBMI, suggested that PF aids in early detection without affecting mortality, but this was contradicted by Loftus et al. [26], who concluded that any type of PF was not an independent predictor of surgical BBMI after multivariate analysis. Our study suggests that PF is significantly more likely in unstable BBMI patients, but significance diminishes when controlling for confounders. PF may not be the primary cause of HI in unstable BBMI patients; contributing factors may include rib-fracture-related chest trauma or intracranial-hemorrhage-related head injury. An investigation into contributors to hemodynamic status between PF and surgical BBMI is warranted. The lower PF incidence (24%) in surgical BBMI patients in smaller studies complicates drawing robust correlations [15]. In our analysis, PF incidence was 12.7%. Future large-scale studies should include more patients with BBMI combined with pelvic fractures and compare the outcomes of different treatment approaches to validate our results. 

Previous evidence has documented that delays of more than five to eight hours for treating surgical BBMI would lead to increased morbidity or mortality [13,14,15], whereas every one-hour delay in TAE for PF-related bleeding would have 1.79 times the odds of in-hospital mortality [3]. Given the different resuscitation locations (operative room or angiosuite), it is difficult to prioritize the management of unstable blunt abdominopelvic trauma patients with the possibility of both retroperitoneal bleeding from PF and intraperitoneal bleeding from BAT, which has troubled trauma surgeons; similar publications have been discussed extensively [1,6,7,11,19,20]. Trauma surgeons must weigh the risks of time-consuming negative pelvic angiography versus the risk of active bleeding in mesenteric tears when planning treatment for patients with BBMI with concomitant PF. Even in the absence of pneumoperitoneum, concomitant mild hemoperitoneum and stable-type simple PF pose difficulty to trauma surgeons because these difficult-to-manage patients usually present a life-threatening condition, with 52–87% unstable hemodynamic and 23–32% mortality rates [6,20]. CT presentation of free fluid without solid organ injuries was a strong independent predictor of BBMI on multivariate analysis in a study by Loftus et al. [26], whereas the size of hemoperitoneum did not indicate a significant peritoneal bleeding requiring therapeutic intervention in patients with PF; even large hemoperitoneum may pose a 30% pseudo-positive possibility in unstable patients with PF without any active intra-abdominal bleeding [6]. In addition, previous studies have mentioned that the outcomes of PF are dependent on the severity of associated injuries and hemodynamics on admission due to the context of multiple traumas rather than the unstable pattern of PF [4]. Even though a simple pelvic ramus fracture might lead to a severe pelvic hemorrhage or an enhancement, CT of the PF without the presence of contrast extravasation cannot entirely exclude arterial bleeding, both of which have been described previously in the literature [2,9,27,28]. As mentioned above, rapid decision making for unstable patients with blunt abdominopelvic trauma despite mild hemoperitoneum and simple PF is challenging for trauma surgeons. 

According to the Advanced Trauma Life Support (ATLS) concept and algorithm, emergency laparotomy is considered the recommended approach for patients with unstable blunt abdominal trauma (BAT) and pelvic fractures (PFs) [29]. Pre-peritoneal packing is a highly recommended laparotomy procedure for achieving temporary hemostasis in unstable patients with pelvic fractures (PFs) whether performed in the emergency department or the operating room. This approach is characterized by its simplicity, speed, and minimal invasiveness when conducted by an experienced surgeon. It serves as a crucial bridge to maintain hemodynamic stability, providing additional time for the preparation of subsequent transcatheter arterial embolization (TAE) if required. Both procedures can be complementary, as supported by the existing literature [1,11,19,20]. However, it is noteworthy that blunt bowel and mesenteric injury (BBMI) remains a less common etiology, necessitating emergency exploratory laparotomy due to active bleeding from bowel injury or disruption of the mesentery, especially in the era of non-operative management. The use of pre-peritoneal packing for PF and peritoneal cavity exploration for BBMI may counteract each other due to the inherent need for cavity exploration, as demonstrated in a prior experimental study [30]. The decision-making process is complex, especially when facing critical conditions. This complexity inspired our motivation to conduct this study, aiming to explore the implications of PF in a cohort of surgical BBMI. The necessity of cavity exploration due to BBMI might impede pre-peritoneal packing, whereas intraperitoneal packing via laparotomy might decrease abdominal pressure and reduce the tamponade effect for retroperitoneal hemorrhage, as documented in an experimental study [30]. However, the decision-making process is complex. A retrospective study by Eastridge et al. [1] found varying mortality rates based on the sequence of angiography and laparotomy, recommending the consideration of transcatheter arterial embolization (TAE) before laparotomy for unstable patients with concurrent hemoperitoneum and unstable PF. In contrast, another study from the Japan Trauma Data Bank (JTDB) found no significant difference in mortality rates, irrespective of the order of TAE and laparotomy, challenging the current guidelines that favor early laparotomy [20]. In our experience, supporting the ATLS algorithm is crucial, as pelvic fractures did not emerge as an independent risk factor for hemodynamic instability (HI) in surgical blunt bowel and mesenteric injury (BBMI) patients. A study by Thorson et al. [11] reinforced this notion, reporting better mortality rates for immediate laparotomy followed by TAE compared to the reverse sequence. Wu et al.’s study [19] suggested post-laparotomy TAE for unstable BAT patients with concurrent PF, especially for those with an injury severity score (ISS) ≥ 16. However, a trauma hybrid operating room is optimal yet uncommon in Taiwan. Facing unstable patients with surgical BBMI and PF, rapid identification of the bleeding source is challenging. Prioritizing laparotomy, coupled with simultaneous preparation for possible subsequent angiography in cases of ongoing HI, should mitigate the risk of morbidity and mortality in the absence of a trauma hybrid operating room. Rather, we believe that our observations will be helpful in assisting timely decision making for difficult-to-manage patients in clinical practice. For a thorough investigation into the impact of various pelvic fractures on the quality of surgical BBMI management, future research is essential. It should involve collecting more cases and examining the management approach according to the severity of different pelvic fractures to validate our findings.

### Limitations

One major weakness, in addition to its retrospective nature and small sample size, was that the main focus of our series was surgically proven BBMI. Due to the small sample size, there may be limitations in statistical power, leading to sampling bias and a potential impact on the results. Secondly, this study only included surgically proven BBMI cases, excluding those unable to undergo surgery or who died before surgery, potentially introducing selection bias. Furthermore, a detailed direct comparison or discussion in patients with concomitant PF was not evaluated, such as TAE first or laparotomy first, quantity of free intraperitoneal fluid, shock duration, resuscitation responders or non-responders, with or without a retroperitoneal contrast brush, or fracture patterns of the pelvis. Lastly, spanning a 13-year enrollment period, this study acknowledges potential variations in treatment and diagnostic approaches over time, which could introduce interference with the results.

## 5. Conclusions

In our study, nearly half of the patients (44%) experienced shock episodes before surgical intervention, demonstrating significantly severe injury severity, critical clinical presentation, and increased morbidity and mortality. This highlights the critical clinical status associated with this issue. Although BBMI patients with pelvic fractures exhibit a significantly higher incidence of hemodynamic instability (HI), those with additional injuries such as rib-fracture-related chest trauma or intracerebral hemorrhage are of greater concern due to the elevated probability of HI. Moreover, while pelvic fractures are significantly more likely in unstable BBMI patients, this significance diminishes when controlling for confounders. This result suggests that, compared to pelvic fractures, BBMI requires more immediate intervention. Therefore, emergency laparotomy is recommended, especially in patients with unstable BBMI with concomitant pelvic fractures, followed by damage control TAE if needed.

## Figures and Tables

**Table 1:** Clinical and injury characteristics of patients with BBMI according to the shock group.

	Overall (N = 158)	Non-Shock (n = 88)	Shock (n = 70)	*p* Value
Age	46.5 (30–59)	48.5 (28–60)	44.5 (33–58)	0.805
Male sex	128 (81%)	75 (85.2%)	53 (75.7%)	0.130
ISS	16 (9–25)	9 (9–18)	21.5 (16–29)	<0.001
ISS ≥ 16	85 (53.8%)	30 (34.1%)	55 (78.6%)	<0.001
ISS ≥ 25	41 (25.9%)	9 (10.2%)	32 (45.7%)	<0.001
NISS	18 (9–27)	13 (9–22)	27 (17–34)	<0.001
TRISS	0.99 (0.938–0.735)	0.99 (0.968–0.994)	0.97 (0.994–0.938)	<0.001
RTS	7.84 (7.108–7.84)	7.84 (7.84–7.84)	7.108 (6.17–7.84)	<0.001
ED vital sign				
SBP (mm/Hg)	116 (91–134)	124 (112–140)	86 (73–123)	<0.001
HR (/min)	96 (80–117)	91 (79–104)	104 (83–126)	0.020
RR (/min)	20 (18–20)	20 (18–20)	20 (17–22)	0.671
GCS	15 (15–15)	15 (15–15)	15 (6–15)	<0.001
Mechanism				
Motorcycle (%)	80 (50.6%)	41 (46.6%)	39 (55.7%)	0.515
Car (%)	42 (26.6%)	22 (25%)	20 (28.6%)	
Fall (%)	3 (1.9%)	3 (3.4%)	0 (0%)	
High fall (%)	6 (3.8%)	4 (4.5%)	2 (2.9%)	
Pedestrian (%)	8 (5.1%)	4 (4.5%)	4 (5.7%)	
Assault (%)	5 (3.2%)	4 (4.5%)	1 (1.4%)	
Bicycle (%)	7 (4.4%)	5 (5.7%)	2 (2.9%)	
Impact (%)	7 (4.4%)	5 (5.7%)	2 (2.9%)	
Clinical presentation				
ED hemoglobin g/dL	12.6 (10.7–14.2)	13.4 (12–14.8)	11.5 (9–12.7)	<0.001
ED intubation (%)	34 (21.5%)	4 (4.5%)	30 (42.9%)	<0.001
Chest tube (%)	31 (19.6%)	11 (12.5%)	20 (28.6%)	0.012
Blood transfusion				
Blood transfusion at ED (%)	85 (53.8%)	22 (25%)	63 (90%)	<0.001
ED pack RBC (U)	2 (0–4)	0 (0–1)	4 (2–7)	<0.001
ED FFP (U)	0 (0–2)	0 (0–0)	2 (0–4)	<0.001
24 HR pack RBC (U)	4 (0–12)	0 (0–4)	12 (8–21)	<0.001
24 HR FFP (U)	2 (0–8)	0 (0–2)	9 (4–16)	<0.001
Massive transfusion (%)	49 (31%)	3 (3.4%)	46 (65.7%)	<0.001
OR pack RBC (U)	2 (0–6)	0 (0–2)	7 (4–12)	<0.001
OR FFP (U)	0 (0–4)	0 (0–0)	4 (2–8)	<0.001
Ward pack RBC (U)	0 (0–4)	0 (0–0)	2 (0–6)	<0.001
Ward FFP (U)	0 (0–4)	0 (0–0)	2 (0–10)	<0.001
Operative finding:				
Isolated bowel injury (%)	41 (25.9%)	38 (43.2%)	3 (4.3%)	<0.001
Isolated colon injury (%)	18 (11.4%)	15 (17%)	3 (4.3%)	0.012
Isolated mesentery injury (%)	48 (30.4%)	15 (17%)	33 (47.1%)	<0.001
Combined injury (%)	51 (32.3%)	20 (22.7%)	31 (44.3%)	0.004
OP blood loss (ml)	500 (100–2000)	100 (50–400)	2000 (1000–3500)	<0.001
Delayed OP	18 (11.4%)	15 (17%)	3 (4.3%)	0.012
Outcome				
Morbidity (%)	106 (67.1%)	43 (48.9%)	63 (90%)	<0.001
Mortality (%)	21 (13.3%)	2 (2.3%)	19 (27.1%)	<0.001
24 h mortality (%)	7 (4.4%)	1 (1.1%)	6 (8.6%)	0.045
Bowel-related mortality (%)	5 (3.2%)	2 (2.3%)	3 (4.3%)	0.656
Exsanguination mortality (%)	11 (7%)	0 (0%)	11 (15.7%)	<0.001
ICU length of stay (day)	3 (2–7)	2 (0–5)	4 (2–14)	<0.001
Hospitalization LOS (day)	17 (11–31)	16.5 (11–30)	18 (9–36)	0.704

BBMI, blunt bowel mesentery injury; ISS, injury severity score; NISS, new injury severity score; TRISS, trauma resuscitation injury severity score; RTS, reverse trauma score; ED, emergency department; SBP, systolic blood pressure; HR, heart rate; RR, respiratory rate; GCS, Glasgow Coma Scale; RBC, red blood cell; FFP, fresh frozen plasma; OR, operative room; OP, operation; ICU, intensive care unit; LOS, length of stay. Data were presented as a number (percentage) and median IQR (25–75%).

**Table 2:** Severity of injury in body regions of patients with BBMI according to the shock group.

	Overall (n = 158)	Shock (−) (n = 88)	Shock (+) (n = 70)	*p* Value
AIS head	0 (0–0)	0 (0–0)	0 (0–2)	<0.001
AIS face	0 (0–0)	0 (0–0)	0 (0–0)	0.413
AIS chest	0 (0–1)	0 (0–0)	0 (0–3)	0.05
AIS abdomen	3 (3–4)	3 (3–3)	3 (3–4)	<0.001
AIS extremities	0 (0–2)	0 (0–2)	0 (0–2)	0.014
AIS head ≥ 2	21 (13.3%)	1 (1.1%)	20 (28.6%)	<0.001
AIS head ≥ 3	15 (9.5%)	1 (1.1%)	14 (20%)	<0.001
AIS face ≥ 2	10 (6.3%)	4 (4.5%)	6 (8.6%)	0.340
AIS face ≥ 3	0 (0%)	0 (0%)	0 (0%)	—
AIS chest ≥ 2	38 (24.1%)	17 (19.3%)	21 (30%)	0.119
AIS chest ≥ 3	35 (22.2%)	14 (15.9%)	21 (30%)	0.034
AIS abdomen ≥ 2	158 (100%)	88 (100%)	70 (100%)	—
AIS abdomen ≥ 3	144 (91.1%)	79 (89.8%)	65 (92.9%)	0.498
AIS extremities ≥ 2	58 (36.7%)	24 (27.3%)	34 (48.6%)	0.006
AIS extremities ≥ 3	28 (17.7%)	12 (13.6%)	16 (22.9%)	0.132

Data were presented as a number (percentage) and median IQR (25–75%). AIS, abbreviated injury score; BBMI, blunt bowel mesentery injury.

**Table 3:** Associated injuries of the patients with BBMI according to the shock group.

	Overall (n = 158)	Shock (−) (n = 88)	Shock (+) (n = 70)	*p* Value
Spleen	13 (8.2%)	6 (6.8%)	7 (10%)	0.470
Liver	29 (18.4%)	13 (14.8%)	16 (22.9%)	0.192
Pancreas	9 (5.7%)	4 (4.5%)	5 (7.1%)	0.511
Urinary bladder	2 (1.3%)	2 (2.3%)	0 (0%)	0.503
Kidney	10 (6.3%)	3 (3.4%)	7 (10%)	0.109
Diaphragm	6 (3.8%)	3 (3.4%)	3 (4.3%)	1.000
Vessel	23 (14.6%)	7 (8%)	16 (22.9%)	0.008
Intracerebral hemorrhage	18 (11.4%)	2 (2.3%)	16 (22.9%)	<0.001
Skull fracture	4 (2.5%)	0 (0%)	4 (5.7%)	0.037
Facial bone fracture	15 (9.5%)	6 (6.8%)	9 (12.9%)	0.275
C-spine	4 (2.5%)	2 (2.3%)	2 (2.9%)	1.000
Lung contusion	18 (11.4%)	7 (8%)	11 (15.7%)	0.127
Rib fracture	27 (17.1%)	6 (6.8%)	21 (30%)	<0.001
Clavicle fracture	10 (6.3%)	2 (2.3%)	8 (11.4%)	0.023
Scapula	3 (1.9%)	2 (2.3%)	1 (1.4%)	1.000
Hemopneumothorax	27 (17.1%)	12 (13.6%)	15 (21.4%)	0.196
Thoracic spine fracture	3 (1.9%)	2 (2.3%)	1 (1.4%)	1.000
Lumbar spine fracture	8 (5.1%)	5 (5.7%)	3 (4.3%)	1.000
Pelvis fracture	20 (12.7%)	5 (5.7%)	15 (21.4%)	0.003
Upper limb fracture	25 (15.8%)	14 (15.9%)	11 (15.7%)	1.000
Lower limb fracture	28 (17.7%)	10 (11.4%)	18 (25.7%)	0.019

Data were presented as a number (percentage). BBMI, blunt bowel mesenteric injury.

**Table 4:** Predictors of shock.

	Univariate Analysis		Multivariate Analysis	
	OR (95% CI)	*p* Value	AOR (95% CI)	*p* Value
Age	1.00 (0.96–1.02)	0.786	—	—
Male sex	0.54 (0.24–1.21)	0.133	—	—
Liver	1.71 (0.76–3.85)	0.195	—	—
Kidney	3.15 (0.78–12.66)	0.106	—	—
Vessel	3.43 (1.32–8.89)	0.011	1.10 (0.32–3.72)	0.882
ICH	12.74 (2.82–57.61)	0.001	10.87 (1.70–69.75)	0.012
Lung contusion	2.16 (0.79–5.90)	0.134	—	—
Rib fracture	5.86 (2.21–15.51)	<0.001	5.94 (1.06–33.45)	0.043
Hemopneumothorax	1.73 (0.75–3.98)	0.199	—	—
Pelvic fracture	4.53 (1.56–13.17)	0.006	2.94 (0.66–13.13)	0.157
Low limb fracture	2.70 (1.16–6.31)	0.022	1.66 (0.51.41)	0.405
Operation finding (compare to Isolated bowel injury)				
Isolated colon injury	2.53 (0.46–13.98)	0.286	—	—
Isolated mesentery injury	27.86 (7.41–104.78)	<0.001	23.50 (4.61–119.88)	<0.001
Combined injury	19.63 (5.34–72.25)	<0.001	25.43 (4.89–132.28)	<0.001

OR, odds ratio; AOR, adjusted odds ratio.

**Table 5:** Incidence rates of complications among patients with BBMI according to the shock group.

	Overall (n = 158)	Non-Shock (n = 88)	Shock (n = 70)	*p* Value
Sepsis	25 (15.8%)	12 (13.6%)	13 (18.6%)	0.398
Pneumonia	24 (15.2%)	9 (10.2%)	15 (21.4%)	0.051
Septic shock	10 (6.3%)	3 (3.4%)	7 (10%)	0.091
Unplanned ventilator	33 (20.9%)	8 (9.1%)	25 (35.7%)	<0.001
Intraabdominal abscess	17 (10.8%)	8 (9.1%)	9 (12.9%)	0.448
Leakage	9 (5.7%)	5 (5.7%)	4 (5.7%)	0.993
Coagulopathy	63 (39.9%)	14 (15.9%)	49 (70%)	<0.001
Acute renal failure	57 (36.1%)	21 (23.9%)	36 (51.4%)	<0.001
Acidosis	46 (29.1%)	11 (12.5%)	35 (50%)	<0.001
Urinary tract infection	26 (16.5%)	11 (12.5%)	15 (21.4%)	0.133
Stroke	4 (2.5%)	0 (0%)	4 (5.7%)	0.023
Pulmonary embolism	2 (1.3%)	1 (1.1%)	1 (1.4%)	1.000
ARDS	5 (3.2%)	1 (1.1%)	4 (5.7%)	0.171
Pleural effusion	25 (15.8%)	12 (13.6%)	13 (18.6%)	0.398
Enterocutaneous fistula	2 (1.3%)	2 (2.3%)	0 (0%)	0.503
Wound infection	33 (20.9%)	18 (20.5%)	15 (21.4%)	0.881
Wound dehiscence	8 (5.1%)	2 (2.3%)	6 (8.6%)	0.140
Abdomen compartment	7 (4.4%)	1 (1.1%)	6 (8.6%)	0.045
tracheostomy	3 (1.9%)	1 (1.1%)	2 (2.9%)	0.585
ECMO	4 (2.5%)	0 (0%)	4 (5.7%)	0.037
Return to OR	24 (15.2%)	9 (10.2%)	15 (21.4%)	0.051
Hemodialysis	3 (1.9%)	1 (1.1%)	2 (2.9%)	0.585

Data were presented as a number (percentage). ARDS, acute respiratory distress syndrome; ECMO, extracorporeal membrane oxygenation; OR, operation room.

## Data Availability

Data were obtained from Chang Gung Research Database and are available by corresponding with the author and obtaining permission.
